# Pylephlebitis as a Rare Complication of Ulcerative Colitis: A Case Report

**DOI:** 10.7759/cureus.4792

**Published:** 2019-05-31

**Authors:** Leonard Hamera, Sunoj Abraham, Jeffrey Jordan

**Affiliations:** 1 Internal Medicine, Citrus Memorial Hospital, Inverness, USA; 2 Pulmonology/ Critical Care, Citrus Memorial Hospital, Inverness, USA; 3 Internal Medicine, Citrus Memorial Hospital / Hospital Corporation of America, Inverness, USA

**Keywords:** pylephlebitis, ulcerative colitis, fusobacterium necroporum, portal vein thrombus, inflammatory bowel disease

## Abstract

Pylephlebitis is a rare and potentially fatal complication of many common abdominal pathologies. Here we describe an unusual case of pylephlebitis associated with ulcerative colitis. A 51-year-old male with a history of ulcerative colitis, diabetes mellitus, and chronic obstructive pulmonary disease (COPD) presented for shortness of breath, fevers, chills, and generalized weakness. The patient was discharged one week prior to this admission after being treated for an acute colitis flare and adverse reaction to sulfasalazine. The current admission was significant for fever, leukocytosis, and lactic acidosis and a portal vein thrombus seen on computed tomography scan. The presentation of sepsis and portal thrombus were consistent with pylephlebitis and he was subsequently started on antibiotics and anticoagulated. He was discharged home and made a full recovery.

Here we present a unique case of pylephlebitis associated with ulcerative colitis. Ultimately this case report serves as a good reminder to stay diligent and keep a broad differential diagnosis when treating a septic patient.

## Introduction

Pylephlebitis, also known as suppurative thrombophlebitis of the portal vein system, is a potential complication of intra-abdominal infections. The incidence has not been well defined in the literature. One study estimated the rate of mesenteric venous thrombosis at 2.7 per 100,000 persons and thus we could postulate the rate of pylephlebitis to be lower [1]. Pylephlebitis can be seen in any age or gender but typically occurs in adult males between 40-65 years of age (60%-70%) [2-4]. Any infection in an organ drained by the portal system can lead to pylephlebitis. Pancreatitis, appendicitis, diverticulitis, cholangitis, and inflammatory bowel disease are commonly mentioned in the literature [3,5,6,7]. There is no one predominating site for thrombosis. The right portal vein, main portal vein, and superior mesenteric vein were each reported around 30% in one study [6]. More recent articles referencing inflammatory bowel disease are referring to Crohn’s disease and, to our knowledge, ulcerative colitis has not been mentioned since 1950 [2,8].

Pylephlebitis commonly presents nonspecifically with malaise, abdominal pain, and fever. Additional history and physical exam findings can include nausea, abdominal pain and tenderness, and splenomegaly. Jaundice and hepatomegaly can also occur and may indicate further complications such as hepatic abscess or cholangitis. Diagnosis requires a clinically suspicious patient with bacteremia confirmed via positive blood cultures and evidence of portal vein thrombus seen either on abdominal computed tomography (CT), ultrasound (US), or magnetic resonance imaging (MRI). Treatment consists of antibiotics tailored to the causative organism plus or minus anticoagulation. Even with modern imaging, antibiotic treatment, and anticoagulation, mortality is estimated between 11%-32% [4,6,9].

## Case presentation

A 51-year-old Caucasian male with a history of ulcerative colitis, diabetes mellitus, and chronic obstructive pulmonary disease (COPD) returned to the hospital, one week following discharge, for acute on chronic shortness of breath, new onset of fevers and chills, and generalized weakness. Previously the patient was seen and treated for abdominal pain secondary to acute inflammatory colitis and sulfasalazine reaction.

Roughly two weeks prior, the patient was started on sulfasalazine for newly diagnosed ulcerative colitis. Shortly after starting this medication he began having increasing abdominal pain, nausea, vomiting, and diarrhea for which the patient was admitted and started on intravenous (IV) fluids and empiric antibiotics. His abdominal CT showed thickening of the wall of the descending colon representing inflammatory changes without additional findings. Serology showed elevated aspartate aminotransferase (AST) 112 IU/L (normal range (NR)=5-35), alanine transaminase (ALT) 312 IU/L (NR=0-55), C-reactive protein 23.72 mg/dL (NR=0-0.50), erythrocyte sedimentation rate (ESR) 101 mm/hr (NR=0-20), and a negative hepatitis panel. Additionally, the patient had a hepatobiliary iminodiacetic acid (HIDA) scan that was negative. A diagnosis of acute colitis secondary to ulcerative colitis was made and an adverse reaction to sulfasalazine was the most likely cause of his elevated liver enzymes. Sulfasalazine was discontinued and the patient was discharged on a prednisone taper with outpatient follow-up.

**Figure 1:**
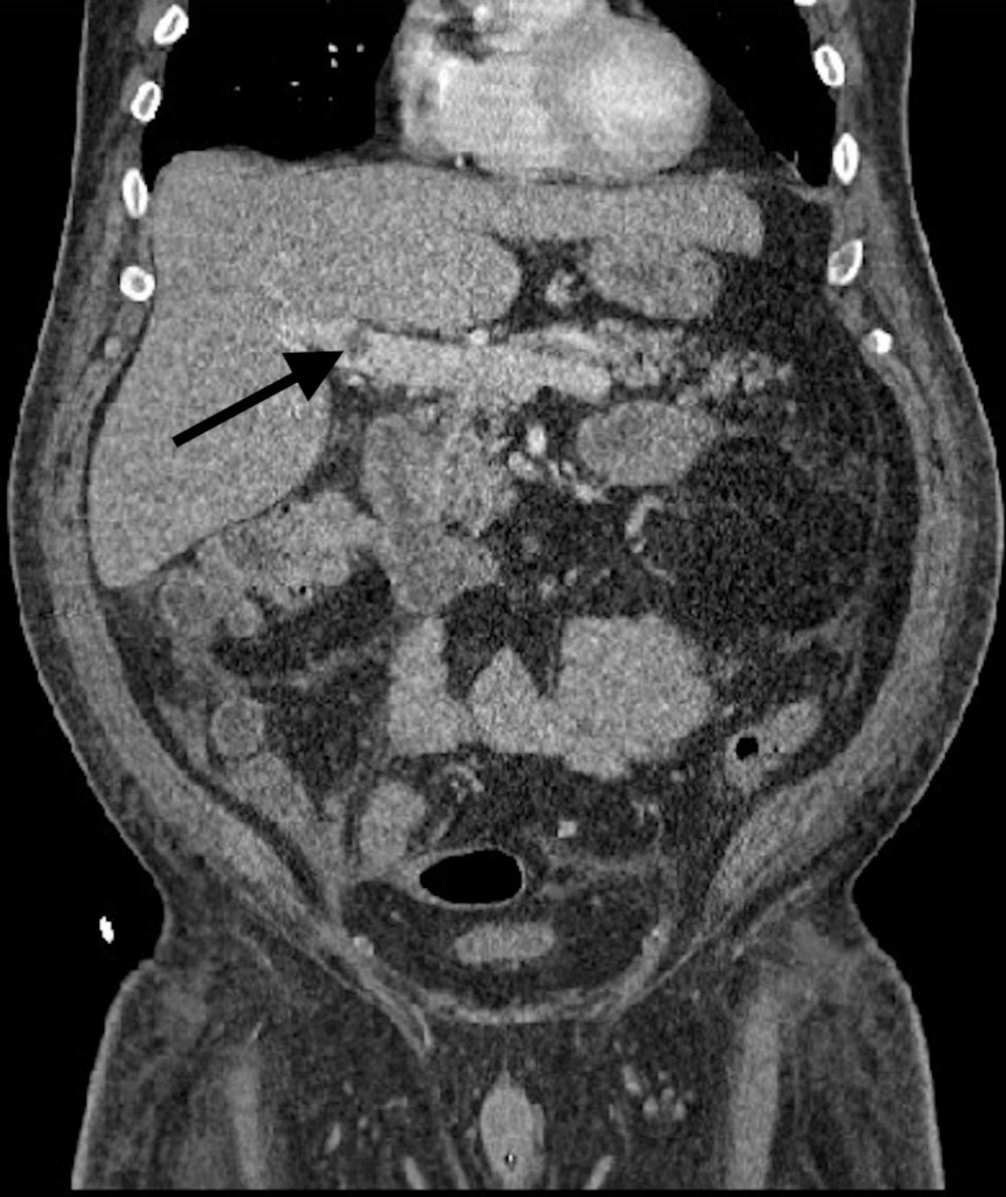
Arrow showing portal vein thrombus.

On the current admission, the patient’s vital signs and physical exam were pertinent for a lethargic appearing male with a core temperature of 104.8 °F, heart rate 156 beats per minute, and respiratory rate of 40 breaths per minute. His serology was significant for an initial WBC count of 4.7x10^3 (85.3% neutrophils), which subsequently rose to 20.2x10^3 (NR=4.5-11), a lactic acid of 6.11 mmol/L (NR=0.5-2.20), AST of 45 IU/L, ALT of 112 IU/L, and direct bilirubin of 1.6 mg/dL (NR=0.2-1.2). The patient triggered our sepsis protocol and was started on 30 mL/kg IV normal saline, had blood, urine, and sputum cultures obtained, and was started on IV meropenem 1g BID and oral doxycycline 100 mg BID. Meropenem was started empirically and doxycycline was added because of suspected tick-borne etiology as the patient had recently traveled to an endemic area. A CT of the abdomen and pelvis was obtained to identify a source of infection. A focal, non-occlusive filling defect in the distal main portal vein just proximal to its bifurcation was seen along with a subtle mass in the medial right hepatic lobe suspicious for abscess not previously seen (Figure 1). Abdominal ultrasound (US) was ordered to investigate a potential hepatic cause given the patient’s elevated liver function tests (LFTs) and confirmed a 1.1 cm main portal vein non-occlusive thrombus at the confluence of the main and right portal veins, but was unable to identify the subtle mass seen on CT. Magnetic resonance imaging (MRI) of the abdomen with and without contrast was obtained and demonstrated cholangitis without abscess.

A diagnosis of pylephlebitis was made. Blood cultures were positive for gram-negative rods and anaerobic rods in 2/2 bottles. Tick-borne disease panel was negative and doxycycline was discontinued. Escherichia coli and Fusobacterium necroporum were isolated from blood cultures and the patient was subsequently switched to IV ceftriaxone 1 g daily and oral metronidazole 500 mg every eight hours, based on sensitivity results, for one month. For anticoagulation he was started on warfarin with an enoxaparin bridge in the hospital to keep his international normalized ratio (INR) between 2 and 3 and continued treatment for three months. The patient showed clinical improvement and was discharged. He had no outpatient complications and was able to make a full recovery.

## Discussion

Currently there are no guidelines for pylephlebitis management. Therefore, diagnosis and treatment plans are based on case series and reports. Imaging is critical in diagnosis as it can point out both the primary source of infection and pylephlebitis. When reviewing the literature we found that abdominal CT, US, and MRI all were able to detect portal vein thrombosis and potential pylephlebitis [3,4,6]. CT is often the initial diagnostic test for non-specific abdominal imaging as it is usually readily available and can rapidly diagnose common intra-abdominal pathology such as appendicitis, diverticulitis, colitis, and pancreatitis. Our initial diagnostic choice was abdominal CT given the patient’s history of UC, but ultimately he received abdominal US and MRI as there was concern for hepatic abscess. In cases like ours where there are elevated LFTs, an abdominal US can help evaluate for hepatic abscess. Other indications of potential hepatic involvement include jaundice, right upper quadrant tenderness, and elevated levels of bilirubin and alkaline phosphatase. If US is inconclusive, MRI can be obtained for further investigation.

Currently no randomized controlled trials have explored the definitive empiric antibiotic regime for pylephlebitis. Therefore initial treatment is with broad spectrum antibiotic coverage based on the likely source of infection and then tailored once culture and sensitivity results become available. Several case series commonly identify polymicrobial infections with E. coli, Bacteroides fragillis, and Streptococcus species [4,6]. Less common organisms identified included Clostridium species, Fusobacterium species, Peptostreptococcus species, and Staphylococcus species [3]. Since blood cultures had grown F. necroporum and E. coli we empirically started meropenem before switching to ceftriaxone and metronidazole based on sensitivity results. While there is no definitive data on the length of antibiotic use, one case review recommended a minimum of four weeks based on a possibility of abscess formation and up to six weeks for known abscess [4]. We chose four weeks since our patient had no evidence of abscess.

Anticoagulation is an area of controversy since no guidelines currently exist. According to the authors of one study there was a benefit observed with the combination of antibiotics and anticoagulation compared to antibiotics alone [8]. The benefits included a lower rate of mortality and recanalization failure. Our choice to anticoagulate was made to help reverse the thrombosis and prevent spread and further complications [3,9]. Although there are inherent risks of anticoagulation such as an increased risk of bleeding, we felt the benefits for our patient outweighed the risks.

## Conclusions

Here we present a unique case of pylephlebitis associated with ulcerative colitis. Ultimately this case report serves as a good reminder to stay diligent and keep a broad differential diagnosis when treating a septic patient.
